# Advances in Sheet Metal Forming Processes of Lightweight Alloys

**DOI:** 10.3390/ma16093293

**Published:** 2023-04-22

**Authors:** Mateusz Kopec, Denis J. Politis

**Affiliations:** 1 Institute of Fundamental Technological Research, Polish Academy of Sciences, Pawinskiego 5B, 02-106 Warsaw, Poland; mkopec@ippt.pan.pl; 2 Department of Mechanical and Manufacturing Engineering, University of Cyprus, Nicosia 1678, Cyprus

With the continuously growing need for more fuel-efficient and sustainable vehicles, the characterization and modeling of metal-forming processes have been indispensable in the development of new products. In the automotive and aviation sector, low-strength structural components are commonly produced from aluminum alloys, and higher-strength structural components are made from ultra-high-strength steels (UHSSs) and titanium alloys. The main issue experienced during the hot forming of complex-shaped components from difficult-to-form alloys is that they are time-, energy-, and cost-intensive. The aircraft industry currently uses methods such as superplastic forming (SPF), superplastic forming with diffusion bonding (SPF-DB), hot stretch forming, hot gas-pressure forming, and isothermal hot forming. Moreover, novel techniques have been developed to produce complex-shaped structural components including solution heat treatment, forming and in-die quenching (HFQ), quick-plastic forming, hot stamping using rapid heating, and fast light alloy stamping technology (FAST).

This Special Issue covered a wide range of topics, including novel materials (HSLA steel [1], titanium alloys [2,3,4,5], magnesium [6,7]), forming techniques (single-point incremental forming [8], magnetic pulse forming [9], rigid-flexible sequential loading forming [10]), and advanced predictive models [1,5,9,11] developed for such processes.

Behrens et al. [1] presented experimental and numerical investigations on HSLA steel friction drilling in which the temperature, strain rate, and rolling direction-dependent tensile tests of the HSLA HX220 were executed and used to parametrize the Johnson–Cook hardening and failure models. Further, the experiments were numerically modelled using different methods. Since the comparison of the simulations and the experiments showed a good agreement, it was assumed that the methods used for the material characterization and modelling were appropriate.

Dang et al. [2] investigated dynamic softening and hardening behavior and microstructure evolution of the TC31 titanium alloy during high-temperature tensile deformation. The authors conclude that the TC31 titanium alloy exhibited clear softening behavior during hot tensile deformation at a temperature of 850 °C and a strain rate of 0.001 s^−1^~0.1 s^−1^, with an increase in the deformation temperature to 950 °C~1000 °C and an increase in the strain rate to 0.1 s^−1^ discontinuous yielding occurred, and quasi-steady flow appeared at a temperature of 950 °C~1000 °C and a strain rate of 0.01 s^−1^, with a decrease in the strain rate to 0.001 s^−1^, resulting in a slight dynamic hardening phenomenon. Furthermore, the authors observed significant microstructural changes when the deformation temperature increased from 850 °C to 950 °C. It was found that the volume fraction of the β phase increased from 20% to 41% after it deformed to a strain of 0.7 with a strain rate of 0.01 s^−1^, whereas the volume fraction of voids was significantly reduced from 11.2% to less than 1%. Since the increased fraction of the β phase at higher temperatures improved the deformation compatibility and reduced the void damage, a relatively high deformation temperature was recommended for the forming of complex TC31 titanium alloy components to avoid void damage. On the other hand, when the TC31 titanium alloy was deformed at 950 °C, the grains grew at the strain rate of 0.001 s^−1^ and were refined at the strain rate from 0.01 s^−1^ to 0.1 s^−1^. The refinement was more significant under the higher strain rate conditions. It was concluded that the appropriate strain rate should be approximately 0.01 s^−1^ during the forming of the TC31 titanium alloy sheet considering both the grain coarsening and uniform deformation.

Li et al. [3] conducted research on the effects of the forming temperature and heating rate on the maximum flow stress and elongation of TC4 by using hot tensile tests and microstructural analysis. It was observed that in the forming temperature range between 850 °C and 950 °C, both the maximum flow stress and elongation decreased with an increase in the forming temperature. When the heating rate was 10 °C/s, the flow stress was larger than that at a heating rate of 1 °C/s, while the elongation remained constant. During microstructural observations, it was found that the volume fraction of the β phase increased with an increase in the heating temperature and a decrease in the heating rate, the average grain size decreased with an increased heating rate and a higher volume fraction of the β phase, and finer grains improved the material ductility. Based on the microstructure observation results, a model was established to predict the volume fraction of the β phase under different heat treatment conditions. The prediction error of the model was 5.17%, which would contribute to a qualitative analysis of the mechanical properties of TC4 titanium alloy under high-temperature deformation conditions.

McPhillimy et al. [4] generated a laser metal deposition tailored preform with a variable thickness to mitigate thinning, a common defect in the room temperature single-point incremental forming (SPIF) of titanium parts with high angled walls. An initial material study of a laser metal deposition (LMD) tailored CP-Ti50A sheet with localized thickening was performed. Subsequently, single-point incremental forming was performed on a LMD tailored CP-Ti50A preform sheet. To facilitate the hybrid LMD + SPIF process, a modular fixture was designed to constrain the tailored titanium sheet during LMD, post-processing, and SPIF.

Su et al. [5] developed an FE method based on the thermal–elastic–viscoplastic macroscopic model to predict the shrinkage, deformation, relative density, and crack of injection-molded Ti-6Al-4V after sintering, using commercially available Simufact software. In the authors’ research, experiments were simultaneously performed to justify the accuracy of the sintering model and simulation method. The results exhibited a good agreement between the experimental measurements and numerical simulations with a 3% error. The slightly larger sintered density and shrinkage in the experiment than those in the simulation were found due to additional thermal convection and conduction during the sintering experiments. It was concluded that the deformation was affected by gravity, friction, specimen shape, and support mode.

Ullmann et al. [6] studied the orientation-dependent flow behavior of the ZAX210 magnesium alloy to provide basic yield model data for the numerical simulation. The authors found that the plane anisotropy Δr was between 0 and −0.2 at all tested temperatures, which indicate a slight anisotropic behavior in the sheet plane. The obtained r-values are a direct result of the crystallographic texture present in the ZAX210 alloy, combined with the relative resolved shear strengths of the slip and twinning systems. The authors conclude that the in-plane material flow behavior can be identified as orthotropic, with decreasing anisotropy at elevated temperatures.

He et al. [7] investigated an unusual phenomenon of strain neutral layer (SNL) spreading revealed during the V-bending test. It was found that the SNL on the middle of the symmetrical surface perpendicular to the transverse direction extends to the compression region with a mound-like boundary. The SNL in the side position was distributed with a parallel band feature. This difference in SNL distribution was mainly attributed to the difference in three-dimensional stress distributions between the side position and the middle position of the bending sample. Finally, it was concluded that the three-dimensional compressive stresses in the compressed region were responsible for the SNL spreading phenomenon.

Yan et al. [8] introduced and optimized a two-stage forming strategy in SPIF to reduce the geometrical deviation and the processing time compared to those manufactured using a single forming tooling. A simulation model of the SPIF has been developed and solved using an explicit finite element analysis to study the optimal tool path for a truncated cone. The design of experiments using a response surface method was used to optimize the proposed two-stage forming strategy. The simulation results showed that the two-stage forming technique could significantly reduce both the geometrical deviation and the forming time. The forming time and part geometric deviation were reduced by 56% and 25%, respectively. In addition, the part thickness distribution was found to be more uniform after optimization, and the minimal thickness decreased by 1.6%.

Mahmoud et al. [9] presented an efficient approach for the simulation of the magnetic pulse forming process of thin sheet metals. The model was developed by combining an electromagnetic solver, relying on Maxwell’s equations, with a mechanical solver, based on the conservation of momentum equations. The overall results showed that the accuracy obtained with a low-resolution SHB approach (a small number of elements) was comparable to that of a high-resolution MINI-element-based technique (a large number of elements). The SHB element was shown to be less stiff than the MINI element. Finally, a computational cost study was carried out, and this demonstrated a higher computational efficiency for the SHB element since a smaller number of elements could be used while maintaining a comparable accuracy to that of the MINI element.

Zhang et al. [10] studied the spring back behavior of large complex multi-feature parts (aluminum alloy inner panel) in the rigid–flexible sequential forming process. Based on the theoretical prediction and experimental results, the spring back compensation of the complex inner panel was carried out. The authors showed that the hardening model has a greater impact on the accuracy of spring back prediction than the yield criterion, and the prediction accuracy of Barlat’89 + Yoshida–Uemori mixed hardening model is the greatest. Finally, the optimized loading locus of hydraulic pressure was obtained, and the accurate results from the compensated parts were used to verify the accuracy of the analysis model.

Liu et al. [11] presented an analytical formability model for the sandwich panel and demonstrated its capability by predicting the critical failure of a sandwich panel consisting of two skin AA5754 layers and a core PVDF layer as a case study. It was found that the developed FLD model overcame the limitation of traditional FLD models and was capable of predicting the formability of sandwich panels made from composite materials. The results presented in this study provided a safety evaluation and theoretical guidance on the plastic-forming and critical failure of the composite sandwich panels for lightweight sealing and insulating component applications.

## Data Availability

Not applicable.

## References

[1] Behrens B.-A., Dröder K., Hürkamp A., Droß M., Wester H., Stockburger E. (2021). Finite Element and Finite Volume Modelling of Friction Drilling HSLA Steel under Experimental Comparison. Materials.

[2] Dang K., Wang K., Liu G. (2021). Dynamic Softening and Hardening Behavior and the Micro-Mechanism of a TC31 High Temperature Titanium Alloy Sheet within Hot Deformation. Materials.

[3] Li D., Zhu H., Qu S., Lin J., Ming M., Chen G., Zheng K., Liu X. (2023). Effect of Heating on Hot Deformation and Microstructural Evolution of Ti-6Al-4V Titanium Alloy. Materials.

[4] McPhillimy M., Yakushina E., Blackwell P. (2022). Tailoring Titanium Sheet Metal Using Laser Metal Deposition to Improve Room Temperature Single-Point Incremental Forming. Materials.

[5] Su S., Hong Z., Huang Y., Wang P., Li X., Wu J., Wu Y. (2022). Integrated Numerical Simulations and Experimental Measurements for the Sintering Process of Injection-Molded Ti-6Al-4V Alloy. Materials.

[6] Ullmann M., Kaden C., Kittner K., Prahl U. (2022). Orthotropic Behavior of Twin-Roll-Cast and Hot-Rolled Magnesium ZAX210 Sheet. Materials.

[7] He C., Liu L., Bai S., Jiang B., Teng H., Huang G., Zhang D., Pan F. (2022). Unusual Spreading of Strain Neutral Layer in AZ31 Magnesium Alloy Sheet during Bending. Materials.

[8] Yan Z., Hassanin H., El-Sayed M.A., Eldessouky H.M., Djuansjah J., A. Alsaleh N., Essa K., Ahmadein M. (2021). Multistage Tool Path Optimisation of Single-Point Incremental Forming Process. Materials.

[9] Mahmoud M., Bay F., Muñoz D.P. (2021). An Efficient Computational Model for Magnetic Pulse Forming of Thin Structures. Materials.

[10] Zhang Y., Lang L., Wang Y., Chen H., Du J., Jiao Z., Wang L. (2022). Spring Back Behavior of Large Multi-Feature Thin-Walled Part in Rigid-Flexible Sequential Loading Forming Process. Materials.

[11] Liu X., Di B., Yu X., Liu H., Dhawan S., Politis D.J., Kopec M., Wang L. (2022). Development of a Formability Prediction Model for Aluminium Sandwich Panels with Polymer Core. Materials.

